# Plantar flexors are the main engine of walking in healthy adults

**DOI:** 10.3389/fspor.2025.1595065

**Published:** 2025-07-08

**Authors:** Viviana Rota, Antonio Caronni, Stefano Scarano, Maurizio Amadei, Luigi Tesio

**Affiliations:** ^1^ IRCCS Istituto Auxologico Italiano, Department of Neurorehabilitation Sciences, Ospedale San Luca, Milan, Italy; ^2^ Department of Biomedical Sciences for Health, Università Degli Studi di Milano, Milan, Italy

**Keywords:** walking, man, muscle power, foot, centre of mass, neural maturation

## Abstract

**Introduction:**

The plantar flexors contribute to the uniqueness of man's walking across bipeds (including apes). This role is achieved in late infancy through neural maturation. This may explain why this mechanism is lost with all corticospinal lesions despite the spared power of plantar flexors in segmental motions. During adult human walking, the plantar flexor muscles at the rear limb, during double stance, are suspected to provide most of the work and power required to translate the body system, which can be represented mechanically by its centre of mass (CoM). However, direct evidence of the dominant role of the ankle muscles in CoM translation is scarce. Experimental evidence requires synchronously assessing the lower limb joints’ and CoM's power.

**Methods:**

In this work, ten healthy adults were requested to walk on a split-belt force treadmill at speeds ranging from 0.3 to 1.2 m s^−1^. A series of eight subsequent strides was analysed at each different speed. The synchronous analysis of ground reaction forces (through force platforms) and joint rotations (through an optoelectronic system) allowed us to simultaneously measure the CoM and the lower limb joints’ power.

**Results:**

The dominant role of the ankle plantar flexors, suggested by previous studies focusing on speeds above 0.9 m s^−1^, was confirmed by observing that changes in ankle power during the push-off phase (end of single stance and initial double stance) mirror the changes in power of the CoM. In the double support phase, the amplitude of the increments in ankle joint power was a strong predictor of the increments in CoM power (*R*^2^ = 82%).

**Discussion:**

Low walking speeds have been included to foster the interpretation of pathologic gaits, and clinical correlates of these findings in motor impairments are highlighted.

**Clinical Trial Registration:**

ClinicalTrials.gov, identifier NCT05778474.

## 1 Introduction

All legged vertebrates, including Man, share basic mechanical characteristics of walking. In fact, despite their different anatomical and neural structures, they share the problem of overcoming gravity and ground shear. The shared solution is saving energy through a pendulum-like conservative motion. During walking, after a foot strikes the ground, the anterior lower limb acts as a lever over which the body system appears to “pole vault” like on an inverted pendulum (1–3). This model was confirmed by studying the motion of the body's centre of mass (CoM), which mechanically represents the whole body (4). Right after the foot strike, the CoM is at its lowest level and decelerates forward; then, it must be re-accelerated and lifted. Consequently, even in the simplest case of straight walking at a constant average speed in the forward direction (steady state walking), the mechanical energy due to the forward speed, the lateral speed, the vertical speed, and the gravitational potential energy of the CoM are continuously changing. The total mechanical energy of the CoM (abbreviated as Etot) is equal to the sum of the forward kinetic energy, the lateral kinetic energy, and the energy due to the vertical motion. This latter is the sum of the vertical kinetic energy and the gravitational potential energy, but it is usually approximated as being equal to the sole gravitational potential energy because, during walking, the vertical kinetic energy is much smaller than the gravitational potential energy. In an ideal pendulum, kinetic and potential energies mirror each other, so Etot is constant. Consequently, no energy input from outside the system is needed to keep it in motion. In walking, however, the energy exchange is not perfect, so Etot undergoes increments sustained by positive muscle work against the ground (so-called positive “external” work, Wext, and “external” power, Ẇext). This injection of muscle work only happens in the “push-off” phase (5) (see below), mostly during its double stance component (the so-called “*a”* increment of Etot), and in the single stance phase (the so-called “*b”* increment of Etot) (6). For a representation of the *a* and *b* increments throughout the stride cycle, see figure 5 in (4).

The “inverted pendulum” mechanics allow a remarkable exchange between kinetic energy and gravitational potential energy. Without this exchange, all the mechanical energy required to accelerate the CoM forward and lift it would come from the muscles. The instantaneous efficiency in the interchange between the kinetic and potential energies of the CoM can be computed as the instantaneous recovery index (7), where 100% stands for complete recovery, i.e., a fully passive translation of the CoM, and 0% stands for motion fully sustained by external (here, muscular) work. On average, across a step, human walking saves up to 60% of the muscular work needed if there were no energy exchange. The extreme values of 0% and 100% are reached for brief periods (8). Although this is not the focus of the present study, it must be recalled that repositioning the limbs at each step also implies muscular work (“internal” Work, Wint), which is not aimed at the advancement of the CoM (9). Additionally, walking implies oscillations also in the left-right direction. Lateral motion implies changes in kinetic energy due to the lateral CoM motion, but such changes are about 5% of the changes in forward kinetic energy (10). Therefore, unless specified otherwise, the lateral motion of the CoM will be neglected in the following text. It is worth noting, however, that the lateral motion of the CoM is highly relevant for balance control (4, 11–15).

As mentioned above, the need for muscle work for CoM displacement arises in two short phases of the step, dubbed the *a* and *b* increments. In *a*, it comes from the loss of vertical energy being insufficient to sustain the needed increments in forward kinetic energy; in *b*, it arises from the decrease of forward kinetic energy being insufficient to sustain the required lift of the CoM. At intermediate and high walking speeds (i.e., from around 0.8 m s^−1^ to 1.6 m s^−1^ in healthy adults), increment *a* is much greater than increment *b*. Looking at power, the peak Ẇext is four times higher in the *a* than in the *b* increment (10). The *a* increment occurs in the so-called “push-off” phase (5, 10), also dubbed as the “step-to-step transition,” which is acknowledged as the step phase requiring the greatest energy expenditure while walking (16). Studies on joint dynamics of the lower limbs during walking demonstrated that, as also suggested by Cavagna (17), the ankle joint of the rear limb provides most of the power during the *a* increment of Etot, and the muscles sustaining this power are, therefore, the plantar flexors. Several studies have investigated the role of the three main joints of the lower limb during walking [for two examples, see (18, 19)], and research has shown that the ankle joint contributes more than half of the individual leg's positive power during walking (20). These findings supported the idea that the plantar flexors are the main engine of human adult walking. However, it is noteworthy that these studies have mainly focused on intermediate to high walking speeds, while slow speeds have usually been neglected. In addition, direct experimental evidence that the plantar flexors dominate the motion of the CoM can be further supported by the simultaneous analysis of limb joints' mechanics and the CoM's motion (5, 21, 22), which is not commonly performed.

The motion of the CoM may help the clinical appraisal of pathological gaits (4). Quite unexpectedly, in studies on pathologic asymmetric walking (caused by various impairments), Wext and Ẇext over a stride (or, equivalently, over a unit distance) may be normal (23–25). Nevertheless, the step performed on the impaired limb presents a saving of muscular work, i.e., a recovery index higher than normal, while the reverse is valid for the unimpaired lower limb. In other words, this reflects the implementation of an adaptive strategy, where the “pole vault” over the impaired limb is nearly passive while the unimpaired limb is overloaded (4, 25–27). It has been suggested that such an adaptation, i.e., the overload of the sound lower limb and the non-use of the impaired one, may hinder any attempts to restore the function of the impaired lower limb, in analogy with amblyopia following strabismus (4, 15, 25). Therefore, it is relevant for both clinical sciences and human physiology to understand how Wext and Ẇext originate at the segmental (joint) level and to identify which muscles contribute to these increments in work and power, as well as the effects of their treatment at the body-system level.

The present study aims to reinforce the existing experimental evidence supporting the plantar flexors' pivotal, though not exclusive, role in body propulsion during walking (5, 21, 22). For this purpose, the relationship between the increments of CoM power and those of ankle joint power has been analysed synchronously at different speeds, including slow ones. In addition, the CoM power has been compared to the combined power of the lower limb's joints throughout the gait cycle. Our experimental hypothesis was that the ankle plantar flexion primarily drives the mechanical power of the CoM during the late single support and the double support phases. Ultimately, the study highlights the uniqueness of human ankle mechanics among walking animals and its clinical significance.

## 2 Methods

### 2.1 Study design

Works from Zelik and coworkers inspired this study (5, 21). The emphasis here is on ankle power during the push-off phase as the primary determinant of body forward propulsion, thereby fostering the clinical interpretation of pathologic gaits. For this reason, low walking speeds have also been tested here, while previous studies focused on speeds above 0.9 m s^−1^ (28).

The data analysed for this study came from an observational cross-sectional study, which was conducted following the Declaration of Helsinki and approved by the ethical committee of the IRCCS Istituto Auxologico Italiano (CLAPENDAS project, Ricerca Corrente IRCCS; protocol code 2019_05_21_01; date of approval 21/05/2019). All participants provided their written informed consent to participate in the study.

### 2.2 Participants

Ten healthy volunteers were enrolled. Participants' enrolment occurred between June 2021 and June 2022 at the Department of Neuro-Rehabilitation Sciences of the IRCCS Istituto Auxologico Italiano in Milan (Italy).

The inclusion criteria were age between 18 and 60, the ability to understand the study's instructions, and the willingness to sign the informed consent form. The exclusion criteria were any neurologic or orthopaedic condition affecting walking or balance, having undergone any major orthopaedic surgery involving the trunk or lower limbs, symptomatic pain conditions, and pregnancy.

### 2.3 Instrumental setting

Walking took place on a split-belt force sensorized treadmill (model ADAL 3D; Médical Développement, Andrézieux-Bouthéon, France) embedded in the floor [for further details on the device, see (29)], located in a dedicated room. The treadmill consisted of two parallel independent half-treadmills, each mounted on four 3D piezoelectric force sensors (KI 9048B; Kistler, Winterthur, Switzerland). In this study, the two half-treadmills ran at the same speed. Force and speed signals were sampled at 100 Hz.

**Figure 1:**
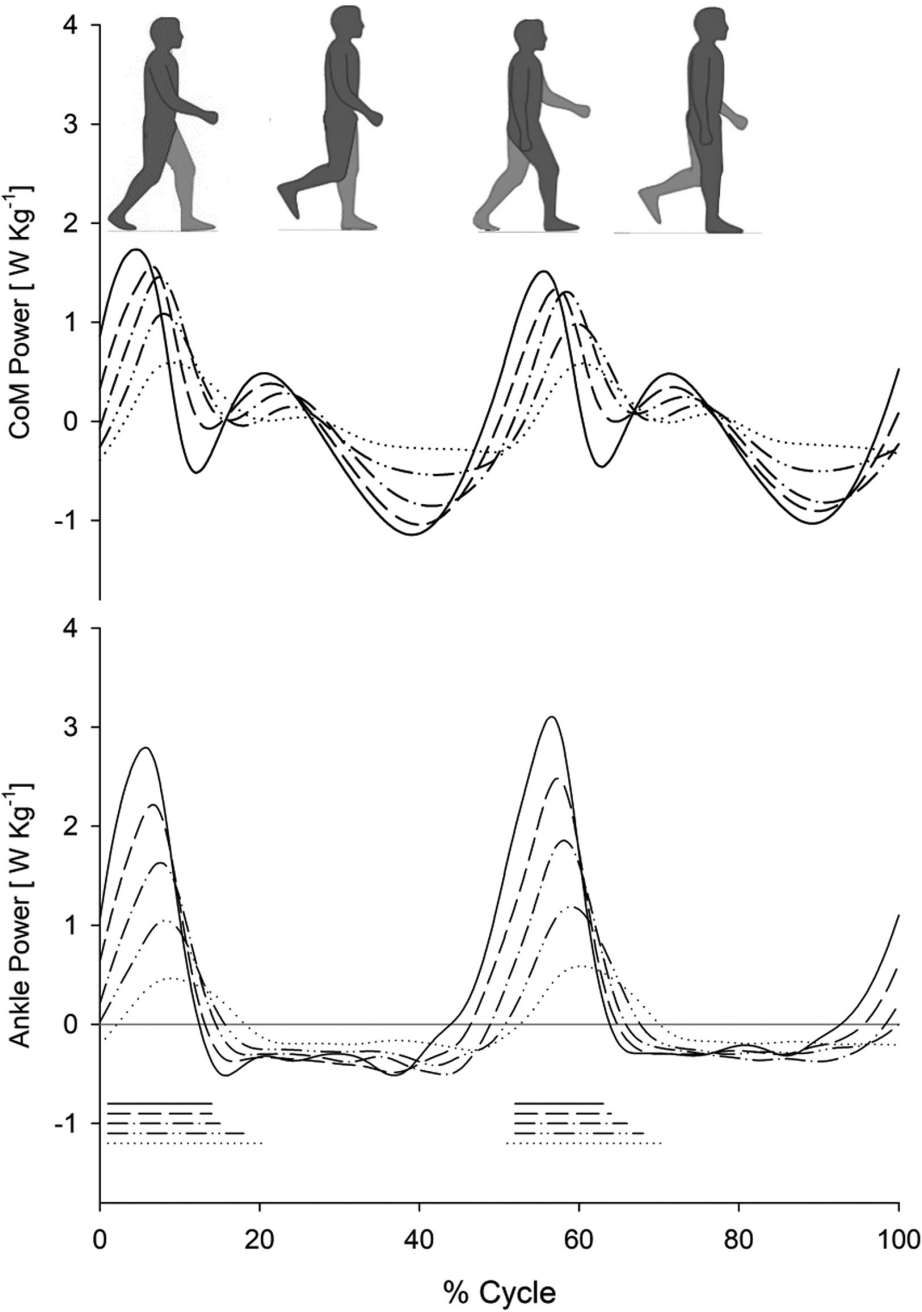
Com power and ankle power during treadmill walking at various speeds. Centre of mass power (CoM Power; upper panel:) and ankle power (lower panel) as a function of the gait cycle (%) at different walking speeds on a treadmill. Each curve represents the average curve from all ten participants who participated in the study at the corresponding speed. The different line types refer to the five speeds described in the plots: 1.2 m s^−1^ (solid line), 1.0 m s^−1^ (dashed line), 0.8 m s^−1^ (dash-dot line), 0.6 m s^−1^ (double dot-dash line), and 0.4 m s^−1^ (dotted line). The mean ± SD stride periods were 1.09 ± 0.06 s, 1.18 ± 0.07 s, 1.30 ± 0.11 s, 1.49 ± 0.13 s, and 1.85 ± 0.24 s, respectively. The horizontal segments at the bottom mark the periods of double foot contact with the ground during the gait cycle (line types recall the corresponding power curves).

Ground reaction forces were synchronised in space and time with the displacement of body markers, hence with joint excursions. Lower limbs' joint kinematics were estimated using an optoelectronic method per the Davis anthropometric model (30). Twenty-one reflective markers were placed on the bony landmarks of each participant's trunk and pelvis and bilaterally on the thighs, shanks, and feet. In details, trunk markers were placed on the spinal process of C7 and the two acromions; pelvis markers were applied on the right and left anterior superior iliac spine and at the base of the sacrum; thigh markers were located on the greater trochanter, on the lateral epicondyle of the femur, and in the middle point between them, for the right and left lower limbs; leg markers were applied in correspondence of the fibular head, over the lateral malleolus, and in the middle point between them, for the right and left lower limbs; foot markers were located, bilaterally, on the posterior surface of the calcaneus and the lateral aspect of the fifth metatarsal head (30) [the setup is illustrated in figure 1 from (29)]. Eight near-infrared stroboscopic cameras (Smart-D optoelectronic system; BTS Bioengineering Spa, Milan, Italy) were used to capture the markers' three-dimensional displacement. Optoelectronic signals were sampled at 100 Hz.

### 2.4 Testing protocol and data collection

The participants were tested for their foot dominance using the revised Waterloo footedness questionnaire (31).

During the study, the participants wore T-shirts, short pants, and light gym shoes to ensure proper positioning and visibility of the reflective markers. After markers were positioned, each participant's height and weight were measured on a precision scale.

Participants were requested to walk on the sensorized treadmill at seven speeds, uninterruptedly and in a single session, as per the sequence: 0.3, 0.4, 0.5, 0.6, 0.8, 1.0, and 1.2 m s^−1^. During walking, the participants were asked to look at a black spot (8 cm diameter) located at eye level on a white wall in front of the treadmill at a distance of approximately 2 m. They were also instructed to keep each foot on the corresponding belt. The participants had to walk freely with no external support. They were closely monitored by two examiners, and a verbal warning was provided before any speed change. For each speed, about 30 strides were requested.

Eight consecutive strides were analysed for each participant at each walking speed, regardless of whether the participant began with the left or right foot-ground contact (see further).

### 2.5 Data analysis

The force platform signals were smoothed using a zero-lag triangular moving average filter. The order of the filter was user-defined and adjusted to match a cut-off frequency of approximately 120 Hz, based on the acquisition frequency and the expected frequency content of the signal. The purpose of this filtering was to reduce high-frequency noise, primarily induced by the mechanical vibrations of the treadmill, while preserving the biomechanical content of interest. This frequency was selected to avoid aliasing and to ensure the accurate calculation of dynamic variables. The same filtering was consistently applied to all signals involved in the computation of CoM power, joint torque, and joint power, following best practices to warrant temporal alignment and prevent filtering-related artefacts (32, 33).

The collected data were recorded using the BTS's SMART Capture software, and specific routines were created *ad hoc* in MATLAB R2021b (MathWorks Inc., Natick, Massachusetts, USA).

Each participant's tracings were visually inspected and divided into individual strides. The stride cycle was defined as the time interval from the foot's contact with the ground (detected by a vertical force exceeding 30 N) to the next ground contact of the same foot. Each stride was then divided into two subsequent steps (the interval between the ground strikes of the two opposite feet). Each step was divided into two phases (single-stance and double-stance phases). Each step was dubbed left or right, depending on the side of the foot striking the ground.

In each step, the Single Stance Phase (SSP) consisted of the interval during which a vertical ground reaction force ≥30 N was recorded under the given limb, only. The Double Stance Phase (DSP) was when a vertical ground reaction force ≥30 N was recorded under both lower limbs, and the given limb was in the anterior position. A DSP occurs twice within each stride cycle (i.e., after each foot initiates contact with the ground), once with the right foot in an anterior position and once with the left foot in an anterior position. Following the prevailing convention, the side of the DSP was dubbed left or right after the side of the limb in the anterior position. Of note, previous research has also adopted the opposite convention, given that the rear lower limb is most influential in gait dynamics (15, 34). Each step's swing phase is synchronous with the single stance phase of the opposite step.

#### 2.5.1 Calculation of mechanical energy transfers and power of the centre of mass

As anticipated in the Introduction, walking can be modelled as an inverted pendulum. In such a model, the CoM undergoes periodic changes in vertical energy and forward kinetic energy (8). The pendular transformation of potential energy into kinetic energy and vice versa reduces the muscular work required to keep the body system in motion relative to the ground, Wext (35).

Implementing Cavagna's procedure, the time course of CoM's kinetic and potential energies was calculated from ground reaction forces (36). In short, the method described by Cavagna begins by measuring, through the force platforms, the vertical and forward components of the reaction force applied to the CoM over a complete stride. The weight of the subject during quiet stance is zeroed, so that only forces above or below weight are considered to obtain the CoM's velocity changes. The vertical and the forward components of the force are used to calculate the accelerations of the body's CoM. Thus, by integrating accelerations, the instantaneous velocities in each plane (both vertical and horizontal) are obtained. The instantaneous kinetic energy is calculated by squaring these velocity components (vertical and horizontal), multiplying each by one-half the body's mass, and summing them to obtain total kinetic energy. The vertical displacement is calculated by integrating vertical velocity as a function of time, and then it is multiplied by body weight to provide the change in gravitational potential energy. As anticipated in the Introduction, Etot can then be obtained as the sum of the instantaneous kinetic and potential energies. Then, the CoM power (CP) was computed as the derivative of Etot with respect to time (4).

#### 2.5.2 Calculation of hip, knee and ankle joint powers

Through the Davis' model (30), hip, knee, and ankle joint powers on the sagittal plane were calculated using data from both force platforms and the position of body markers detected by the optoelectronic system.

This approach models the human body as a series of rigid segments connected by joints. The joint moment is estimated through the vector product of the distance from the joint rotation (horizontal) axis, estimated through the Davis model, to the point of application of the resultant force. Joint power is then determined by multiplying the joint moment by the joint's angular velocity. Thus, the powers of the right and left ankle joints (Ankle Power, AP), knee joints (Knee Power, KP), and hip joints (Hip Power, HP) have been estimated. Then, the sum of HP, KP, and AP (hip-knee-ankle power, HKAP) was calculated for the right and left lower limbs, respectively.

#### 2.5.3 Calculation of power increments and power peak latencies

In the present analysis, each power trace — including CP, AP, and HKAP — has been examined during the SSPs and DSPs of right and left strides, respectively.

For CP, AP, and HKAP, each trace's minimum and maximum values were identified separately within the SSP and DSP phases. Then, the power increments (i.e., power maximum – minimum) were calculated. The latency of the power increment corresponded to the latency of the maximum (i.e., the power peak) from the stride beginning. It is worth noting that the minimum values could be negative, indicating that joint power was absorbed rather than generated (a stretch-shortening cycle of active muscles is implied).

### 2.6 Statistics

Linear mixed-effects models (LMMs) (37) have been used for the statistical analysis, and values from each individual stride have been entered for the analysis (full random intercept and random slopes model). The gait speed (continuous variable) and the side (i.e., dominant vs. non-dominant lower limb; categorical variable) were incorporated as fixed effects, while the participants (categorical variable) and limb side were included in the models as random effects. The Akaike Information Criterion (AIC) (38, 39) was used for model selection. In case of an absolute AIC difference >4, the model with the smallest AIC was preferred. Models with an absolute AIC difference <4 were considered equivalent, and, in this case, the simplest model was further considered (40). More precisely, the AIC was used to compare a full model that included an interaction term with a simpler one without interaction. Note that the latter is nested in the former.

For hypothesis testing, ANOVA was calculated for the fitted models. More precisely, the Analysis of Covariance (ANCOVA) was performed because models included gait speed, i.e., a continuous variable, as one of the predictors. Degrees of freedom were calculated using Satterthwaite's method.

When regression models included only a continuous predictor and a continuous response variable (e.g., the amplitude of the AP and CP increments, respectively), *t*-values were calculated from the regression estimates and their standard error for hypotheses testing. Similarly to before, degrees of freedom were computed according to Satterthwaite. The significance level was set at 0.05.

Regarding the regression assumptions, the normality of the residuals and the homogeneity of their variance were visually verified. In case these assumptions were violated, the response variable was transformed. For better understanding, the graphs reported the data as untransformed.

The sample size was based on previous studies (15, 28, 41–43). A sample size of 10 participants was deemed sufficient, as the experimental conditions tested here provide high reproducibility of the results, thanks to the known and constant walking speed imposed by the treadmill (29).

Demographic data on the participants were summarised using median and range.

MATLAB^TM^ software was used for analysing the signals. All the statistical analyses were run in R (44). R, MATLAB and Microsoft PowerPoint^TM^ were used for figure plotting and editing.

## 3 Results

### 3.1 Participants

A sample of ten healthy adults (four females) was recruited, with a median (range) age of 28.5 (21–42) years, height 1.72 (1.62–1.81) m, weight 73.4 (51.7–93.2) kg, and BMI 24.9 (18.5–29.7) kg m^−2^. Six participants had a right-dominant foot, while four were left-dominant.

All participants were able to complete the walking task at the requested speeds. No interruptions were ever asked, and no adverse events (e.g., stumbling or falling) occurred.

### 3.2 Analysis of power increments and peak latencies of AP, CP, and HKAP as a function of side and of gait speed

First, a preliminary analysis was conducted to determine how the amplitude of power increments and the power peak latencies of CP, AP, and HKAP were related to gait speed. Asymmetries between strides named after the dominant and non-dominant lower limbs were also assessed. In this analysis, the dynamic variable (i.e., CP, AP, or HKAP) was incorporated in the model as the dependent variable.

#### 3.2.1 Speed-related changes of CP increment amplitude and latency

A positive, significant relationship was found between the increment of CP and gait speed for both lower limbs in DSP (F1, 1,031 = 3,495.1, *p* < 0.001) and SSP (F1, 948 = 1,211.1, *p* < 0.001).

In addition, in the DSP, the slope of this relationship was different for the phases dubbed after the anterior, dominant or non-dominant lower limb, as indicated by the interaction between side and gait speed (F1, 1,036 = 13.7, *p* < 0.001). It must be recalled that this convention obscures the fact that the rear lower limb provided the power needed in this phase.

The slope was steeper for the “non-dominant” DSP (which means it was steeper when the rear, dominant lower limb provided most of the muscle power needed). However, it must be stressed that this slope difference between the two sides was negligible (dominant side: *β* = 2.24; non-dominant: *β* = 2.51). To appreciate the effect size of the difference between the two sides, at 1.2 m s^−1^, one has to consider that the CP increment was 3.24 and 3.08 W Kg^−1^ for the DSP dubbed after the non-dominant and dominant lower limbs, respectively. Therefore, given an average CP increment of 3.16 W Kg^−1^, the difference between the two lower limbs was about 5% of this value.

A significant relationship was found between gait speed and the latency of the CP increment in the DSP (F1, 1,031 = 612.2, *p* < 0.001) and SSP (F1, 949 = 791, *p* < 0.001), with no difference between the two sides. In particular, the higher the speed, the earlier the CP positive peaks (DSP: *β* = −4.77, *p* < 0.001; SSP: *β* = −7.29, *p* < 0.001).

#### 3.2.2 Speed-related changes of AP increment amplitude and latency

With increasing gait speeds, the amplitude of the increments of AP in the DSP increased at both lower limbs (F1, 1,032 = 5,447.41, *p* < 0.001). No side asymmetries were found for AP increments in DSP.

Regarding the latency, the higher the speed, the earlier the peak of the AP increment in the DSP (F1, 1,032 = 331.59, *p* < 0.001). Again, no side asymmetries were found for AP latencies.

#### 3.2.3 Speed-related changes of HKAP increment amplitude and latency

With increasing gait speeds, the increment in HKAP in the DSP became larger for both lower limbs (F1, 1,020 = 7,170.98, *p* < 0.001), with a steeper slope for the non-dominant lower limb (*β* = 4.66) than the dominant one (*β* = 4.25); interaction between side and gait speed: F1, 1,021 = 17.01, *p* < 0.001). Similarly to the CP increment, the increment of HKAP in the DSP was larger for the non-dominant lower limb at higher gait speeds and smaller at lower speeds. However, although significant, the difference between lower limbs in the steepness of the relationship between gait speed and the power increment was negligible.

Likewise, the increment in HKAP in the SSP became higher for both lower limbs with increasing gait speeds (F1, 605 = 692.66, *p* < 0.001). In this case, the steepness of the relationship between gait speed and the increment was lower for the non-dominant (*β* = 1.05) than the dominant limb (*β* = 1.44; F1, 606 = 6.29, *p* = 0.012).

The higher the speed, the earlier the HKAP peaks in both the DSP (F1, 1,020.78 = 528.03, *p* < 0.001) and SSP (F1, 617.23 = 820.96, *p* < 0.001). The two DSP phases showed no asymmetries between the two sides.

In summary, the speed-dependence of both power increments and peak latencies was consistent across CoM and joint dynamics. The overall asymmetries were minor and were thus neglected in further modelling.

In Figure 1, tracings representing CP (upper panel) and AP (lower panel) during the gait cycle (two subsequent steps) at five different gait speeds (0.4 m s^−1^, 0.6 m s^−1^, 0.8 m s^−1^, 1.0 m s^−1^, and 1.2 m s^−1^), for the entire sample of ten participants (average curves from all ten participants), are reported. The increase in CP and AP increments, along with the reduction in CP and AP peak latencies, can be observed in the tracings as gait speed increases.

**Figure 2:**
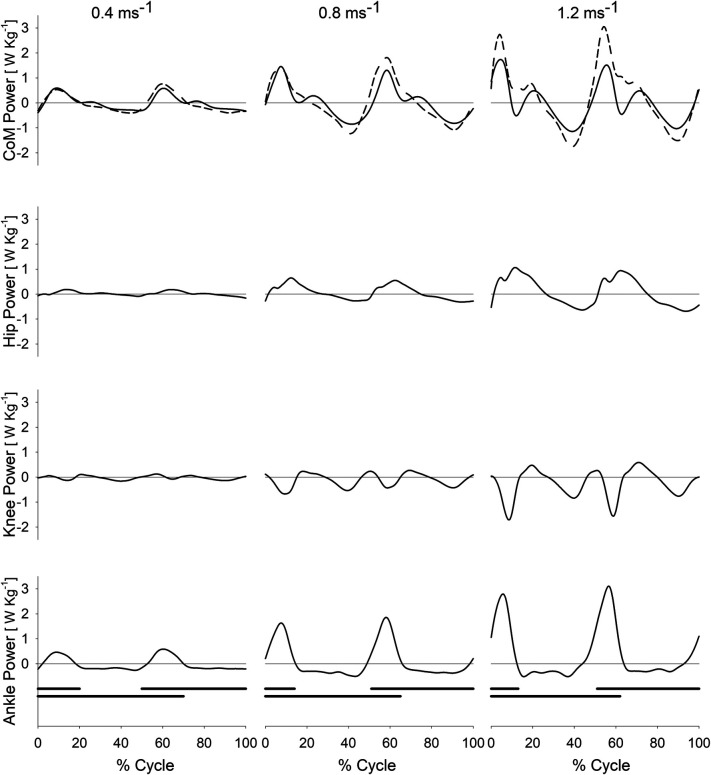
CoM power and joint powers of the hip, knee and ankle during treadmill walking at different speeds. Centre of mass power (CoM Power, CP; first row from top, solid line), summed powers of the hip, knee, and ankle joints (Hip-Knee-Ankle Power, HKAP; first row from top, dashed line), hip power (second row), knee power (third row), and ankle power (fourth row) as a function of the gait cycle (%, abscissa) at different walking speeds on a treadmill (0.4, 0.8, and 1.2 m s^−1^ in the leftmost, middle and rightmost column, respectively). Each curve represents the average curve from all ten participants at a given speed. The HKAP curve has been obtained for each participant by summing the ankle, knee and hip curves at each instant. The black horizontal bars at the bottom of each column mark the periods of foot contact with the ground during the gait cycle. The double stance phases are identifiable by the overlap between the two black bars.

In Figure 2, the individual joint powers (AP, KP, and HP), as well as the HKAP and CP, during the gait cycle, are illustrated for three different walking speeds (0.4 m s^−1^, 0.8 m s^−1^, 1.2 m s^−1^) for the entire sample of ten participants (average curves from all ten participants). Note the difference between the values of CP (solid line) and HKAP (dashed line) in the uppermost row.

Of interest is that all joints, including the ankle, absorb energy (see the negative power values) during the mid-SSP, coinciding with a decrease of CP. This indicates that the plantar flexors and the knee and hip extensors of the pivoting limb, known to be active during elongation, exert a braking action during the single stance, in particular during the fall and forward acceleration of the inverted pendulum.

### 3.3 Graphic representation of the ankle-CoM power increments relationship in the double support phase

This analysis investigated the increments of CP occurring in the DSP as a function of the increments of AP in the same phase. The study aimed to determine the role of AP power increments in “fueling” the CP, hence whole-body translation. Therefore, CP was incorporated in the model as the dependent variable, while AP was introduced as fixed and random effects.

**Figure 3:**
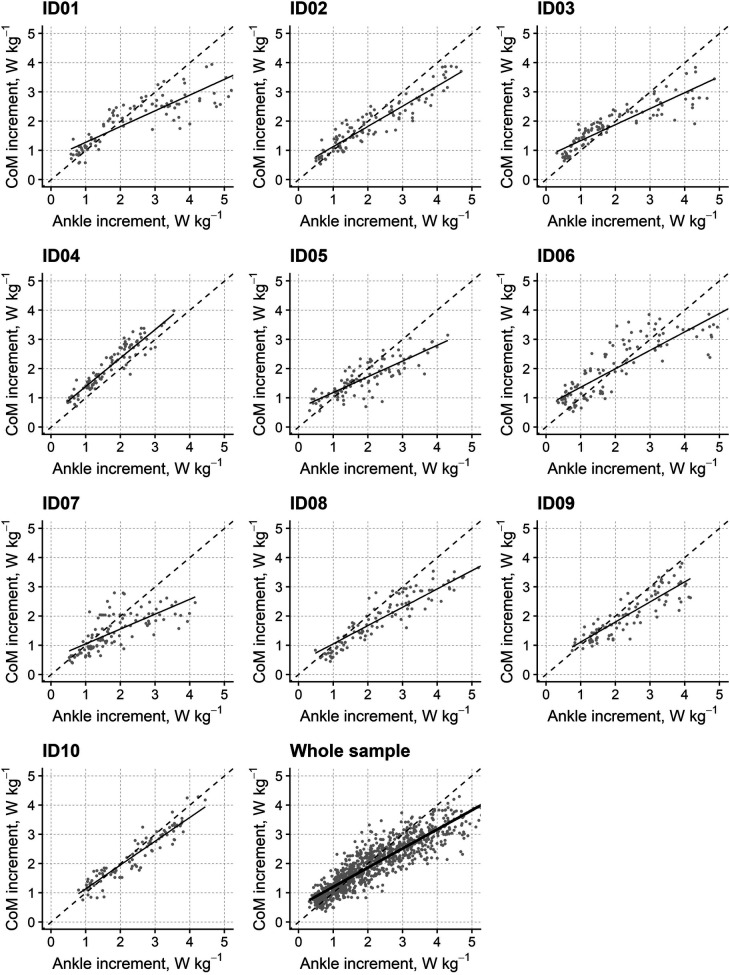
Relationship between the increments in CoM power and ankle power for individual participants and the whole sample in the double stance phase. The graphs report (*y*-axis) the increments of the CoM's power as a function of the increments in ankle power (*x*-axis) occurring in the double support phase at different walking speeds for the ten individual participants (“ID01” to “ID10”) and for the whole sample (“Whole sample”). Solid lines represent the regression lines from linear mixed-effects models, while grey dots refer to individual strides at different speeds. The identity line (i.e., bisector; dashed line) was added as a visual guide.

Figure 3 shows the relationship between the AP and the CP increment for each of the ten participants and the whole sample (with each dot representing a single stride). The AP increment was a significant predictor of the CP increment (estimate: *β* = 0.656; 95% CI: 0.556–0.757; *t*-value = 14.16, d.f. = 9.34, *p* < 0.001) and explained up to 82% of the CP increments' variance. Figure 3 also highlights that the regression line consistently has a lower slope (see above) and an offset (intercept: 0.547; 95% CI: 0.412–0.675) with respect to the identity line. This finding could suggest that there are significant sources of CoM power in addition to the AP at lower walking speeds. Conversely, not all the AP is applied to the CP at higher speeds.

### 3.4 Relationship between the lower limb joints and CoM in terms of power increments amplitudes and latencies in the double support and the single support phases

**Figure 4:**
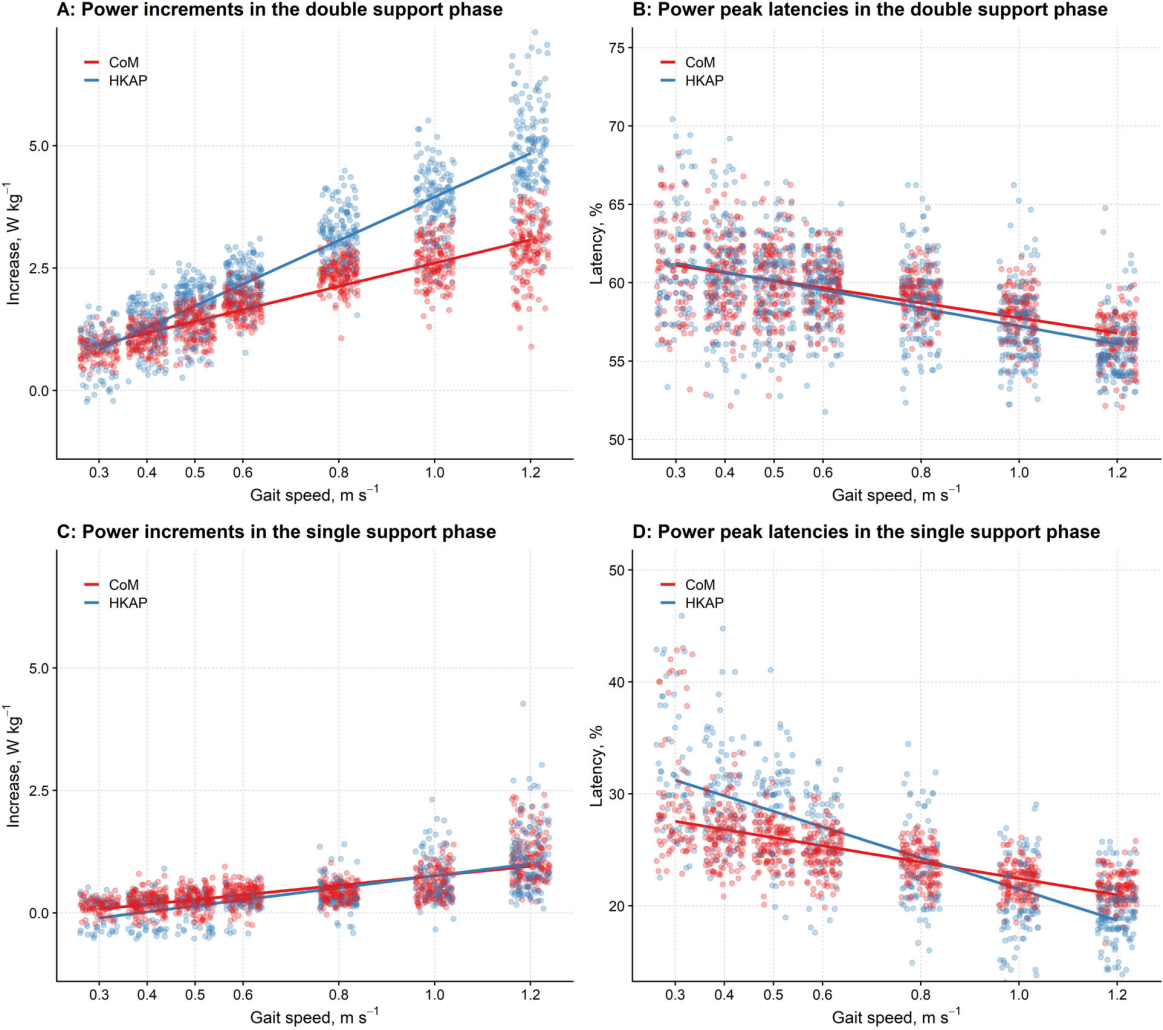
The differences in the power increments and peak latencies between the CoM's power and the combined powers of the hip, knee, and ankle joints at different gait speeds in the double and single support phases. **(A,C)**: The graph reports (*y*-axis) the increments of the CoM's power (CP, red) and the summed powers of the hip, knee, and ankle joints (HKAP, blue) to the gait speed (*x*-axis) in the double support phase **(A)** and the single support phase **(C)** Regression lines from linear mixed-effects models with gait speed and CP increments vs. HKAP increments as predictors are given by dots representing partial residuals (45) of individual strides. For both the CP and the HKAP, the higher the gait speed, the larger the power increase. However, in the double support phase, the regression slope is significantly higher for HKAP, so HKAP is more remarkable for high gait speeds. Conversely, no difference is apparent for very low – low gait speeds (i.e. < 0.4 m/s). In the single support phase **(C)**, the slope of the regression line is more prominent (i.e., the rate of increase is greater with increasing speeds) for HKAP compared to CP; at the walking speeds tested here, this difference was slightly more evident for slower speeds. **(B,D)**: The graph reports (*y*-axis) the latencies of the CoM's power peak (CP, red dots) and the summed powers of the hip, knee, and ankle joints (HKAP, blue dots) to the gait speed (*x*-axis) in the double support phase **(B)** and the single support phase **(D)** Regression lines from linear mixed-effects models with gait speed and CoM latencies vs. HKAP latencies as predictors are given with dots representing partial residuals of individual strides. For both the CP and the HKAP, the higher the gait speed, the earlier the peaks in the double support phase **(B)** and the single support phase **(D)** As shown in B, in the double support phase, the latencies of CP and HKAP are superimposable for low gait speeds, while at higher speeds, the peak of HKAP occurs earlier than CP. However, this difference looks negligible at the walking speeds tested here, and it is more evident in the single support phase **(D)**, where the slope of the regression line of HKAP shows a steeper reduction. Horizontal jittering has been used in the graphs to reduce data points overlapping.

Figure 4 shows the relationship between gait speed and the increments of CP and HKAP, i.e., the point-by-point sum of the ankle, knee, and hip powers. Data come from the regression model with power increment as the response variable and gait speed, the type of signal (CP vs. HKAP), and their interaction as predictors. Individual dots represent the partial residuals (45) of the different strides.

As shown, the higher the gait speed, the larger the power increment in the DSP (F1, 2,070 = 9,072.23, *p* < 0.001). However, as the significant interaction pointed out (F1, 2,071 = 428.60, *p* < 0.001), the slope of this increase was significantly different between the two signal types, being steeper for HKAP than CP (Figure 4A).

Similarly to the DSP, the power increment for the SSP increased with gait speed (F1, 1,573 = 1,728.58, *p* < 0.001), with a slope significantly differing between the two signals (F1, 1,573 = 18.22, *p* < 0.001), which was higher for HKAP than CP (Figure 4C). However, although statistically significant, the difference between the two slopes is lower than that for the DSP. It is also worth noticing that, in the SSP, a difference between the two methods is apparent only for low gait speeds; at higher speeds, the two methods return superimposable amplitudes.

Regarding the latency of the peak of the power increment in the DSP, the higher the gait speed, the earlier this peak (F1, 2,071 = 915.20, *p* < 0.001), more markedly for the HKAP curve (Figure 4B) than for CP (F1, 2,078 = 7.20, *p* = 0.007).

A similar, although much stronger, relationship was found for the increment latency in the SSP: the higher the gait speed, the earlier the peak (F1, 1,574 = 1,690.98, *p* < 0.001), more sharply for the HKAP (Figure 4D) than the CP curve (F1, 1,567 = 165.05, *p* < 0.001).

## 4 Discussion

### 4.1 Limitations

The limits of the present paper should be highlighted first. Above all, the sample is modest, and tests were conducted on a treadmill, not on firm ground. Concerning the former point, it must be acknowledged that the reduced sample size could affect the generalizability of the study's results. However, the sample size of the current study is comparable to those from previous research (15, 28, 41–43). It is important to note that walking on the treadmill, imposing a known and constant speed, makes the tests highly reproducible, thus minimising variability within and between subjects. Therefore, from a statistical perspective, this high reproducibility could hopefully compensate for the low sample size. However, in future studies aiming to develop a normative database of gait parameters (46) for treadmill walking, researchers should consider recruiting larger samples. Concerning the latter point, the differences between treadmill walking and ground walking have been widely investigated and discussed in the literature [for two recent systematic reviews, see (47) and (48)]. Despite some inconsistent findings between individual studies, the main kinematic difference between the two contexts seems to be a reduction in stride length and an increase in step cadence (48), for any given walking speed. This difference has a negligible impact on the mechanical energy changes of the CoM (49). In addition, the relative oxygen consumption is increased, and the vertical ground reaction forces at push off are lower (48), consistent with the shorter steps. For these reasons, although motorised treadmills provide an invaluable tool for the controlled manipulation of locomotor tasks, literature reviews usually recommend care when generalising results to overground walking. From the perspective of the present study, it is essential to highlight that a recent systematic review and meta-analysis did not report significant differences between treadmill and overground walking in terms of the moment and power of the plantar flexors (48). In addition, a previous study comparing the same sensorized treadmill used here with data from healthy adults walking overground did not show significant differences for dynamic and kinematic parameters or muscle electromyographic activities (29).

One further limitation is that only low and intermediate walking speeds were explored. On the treadmill, higher speeds are rarely possible (and even less likely to be affordable). In pathological gaits, this limitation seems of little relevance to clinical considerations. However, further studies are needed to clarify how impairments involving the plantar flexor muscles can affect the CoM's mechanics in pathologic gaits [for a rare example, see (15)].

Moreover, the statistical analysis performed in the present study did not take into account the individual powers of the hip and knee joints, which were only considered as part of the total HKAP. However, relevant research from other groups has provided details on the individual contribution of the three main joints of the lower limb during walking and running (20, 50).

### 4.2 Findings from the dynamic analysis

The present study's results essentially replicate those of Zelik et al., with some relevant extensions. Therefore, the study adheres to the request for independent confirmation of results in published papers (51).

The dominant role of the plantar flexors in body propulsion during human walking, increasing with speed, has been suspected for a long time. Cavagna et al., for instance, highlighted that the increment of Etot implies a growing need for plantar flexion, allowing an increase in step length once the hip range of extension is saturated (thereafter, only a cadence increase is possible) (17). The overall picture suggests a role for the plantar flexors of the rear limb during the double stance phase in ensuring the body system's propulsion. During the double stance, the ankle power curve (Figure 1 and Figure 2, bottom rows) nearly mirrors the power curve of the CoM (uppermost rows; solid line in Figure 2). Figure 2 shows that the scarce positive (extensor) power at the hip adds very little (see HKAP in the uppermost row, dashed line). Regarding the knee, no extensor power is generated at that point in the gait cycle. However, at the highest speeds, total ankle, knee, and hip power (i.e., HKAP) exceeds the CoM power, a finding already noted by Zelik. The time course of CP is markedly different from the time course of total joints' power (HKAP) during single stance (Figure 2). Positive power is produced at the CoM level (the direct consequence of Cavagna's *b* increment), the more, the higher the speed. This increment can be attributed to positive (extensor) power at the knee and the hip. Our results are consistent with previous evidence from Montgomery & Grabowsky analysing the contribution of individual lower limb joints during walking over a range of speeds and at different slopes. The Authors confirmed that the ankle joint provides a large portion of the propulsive power moving the CoM during the double stance. They reported that the ankle and hip contribute 51% and 33% of the individual leg's positive power, respectively (20). Notably, this study requested participants to walk on a dual-belt instrumented treadmill at speeds ranging from 1.0 to 1.5 m s^−1^. Thus, our data can be considered to complement their results for lower walking speeds.

The role of the plantar flexors in human walking is not given the appropriate prominence in clinical observation. Larger muscles (i.e., quadriceps, glutei, hamstrings) seem more suitable candidates to represent the main walking engine. Different authors highlighted the role of plantar flexors as brakes or propellers. On the “braking” side, these muscles are active (while elongating) during the fall of the CoM in the mid-phase of the single stance (see Figure 2). The hip and knee extensors seem to play a similar role. Indeed, according to some Authors, the contribution of the plantar flexors to whole-body forward displacement would mainly consist in restraining forward tibial rotation (i.e., stabilising the knee and ankle joints) (52), and in “braking” the CoM's falling (53). As demonstrated by Honeine, adding a load to the body (about 30% of body weight) does not affect the EMG activity of the triceps during push-off, thus supporting a “stabilising” role of these muscles (43). On the “propulsion” side, other authors emphasised the role of the plantar flexors in providing the so-called push-off during active shortening (18, 54–57), i.e., in “fueling” the body's propulsion. Hence, our results, as displayed in Figure 3, showed that the increment in ankle power faithfully parallels the increment in CoM power (Figures 1, 2). Therefore, our findings support the conclusions of Zelik et al., according to which the primary contribution of plantar flexors at push-off is sustaining the progression of the CoM and the leg's swing (5). On the other hand, the “braking” role of the plantar flexors is by no means incompatible with their propulsive role, in distinct phases of the step.

The role of the hip and knee deserves some consideration. It may appear counterintuitive that the bulky muscles acting on the hip and knee play a secondary role in body propulsion. A relevant outcome of Zelik's work was a “unified perspective of ankle push-off in human walking” (5). As previously discussed, the ankle propulsive role has received various interpretations in the literature, sometimes only based on a kinematic, not dynamic, analysis: these were, for instance, facilitating the roll-over on foot, smoothing the trajectory of the CoM by lowering its vertical lift and, of relevance here, providing the advancement of the CoM (hence Ẇext) as opposed to providing the lift and swing of the rear limb (typically seen as sustained by “internal” power). Zelik and Adamczyk emphasised that these opposing views are not mutually exclusive. The power spent to accelerate and lift the lower limb (which has a non-negligible mass) is found in the final count of CoM power. In cyclic conservative movements (like the ideal pendulum oscillations), the average overall energy of the motion of the body system is mainly related to the overall body mass. Still, in non-perfectly conservative human movements (such as walking), the necessary energy increments sustained by muscle power can be influenced by energy changes of segments [see the enlightening example of the child on a playground swing (5)]. Therefore, thigh lifts through hip flexion may contribute to Ẇext, not only to “internal” power.

The “excess” power generated by the ankle, compared to the power recorded at the CoM level during the double stance, might be spent to overcome soft tissue deformations [see figure 6 in (42)]. According to another compatible perspective, it might be paid to compensate for the negative power the leading leg produces at ground collision. The double integration method only allows us to “see” the motion (including its power) of the CoM, which is not influenced by the positive power of single joints working against each other. Highlighting the perspective of lower limb power, the former approach was dubbed by Donelan et al. “combined limbs method”, while the latter was dubbed “individual limbs method” [see figure 2 in (58)].

### 4.3 Clinical considerations

The clinical relevance of these findings deserves some consideration. First, the speed range explored fits with clinical observations. Pathologic walking rarely attains speeds higher than those observed in the present study. This finding holds with greater force in studies on subjects walking on a treadmill (both adults and children), like in the present study and Zelik's studies. While on treadmills, subjects tend to adopt spontaneous speeds lower by about 30% compared to those adopted on firm ground, with a step length about 9% shorter than ground walking for the same speed (15, 59). The same holds for the average speed of the CoM during walking on split-belt treadmills imposing different speeds on each lower limb (41, 60, 61). Second, most forms of pathologic limping in brain or spinal cord impairments are characterised by a loss of plantar flexors' power (see below). This seems not to be the case when limping is simulated in healthy subjects walking on a split-belt treadmill (41). Compared to the lower limb, the faster limb has a shorter stance (like in truly pathologic limping), but it is “dragged” behind by the speedier belt and needs more power to re-align with the slower limb. When belts run at 0.8 and 0.4 m s^−1^, respectively, the ankle on the faster belt provides a peak power nearly five times higher than the opposite ankle and 1.2 times higher than the power provided when both belts run at 0.6 m s^−1^. There are no power asymmetries at the knee, and -paradoxically- there is a 25% lower peak extensor power at the hip on the faster compared to the slower side (60). In short, when gait is artificially made asymmetric in healthy subjects, the plantar flexors again emerge as the main walking engine.

By contrast, in hemiplegic walking, the hip, knee, and ankle on the paretic side all provide lower extensor (plantar flexor) power than the homologous joints on the unaffected side. The asymmetry is highest for plantar flexion (62). The power asymmetry between lower limbs also characterises “crouched” (flexed-hip, flexed-knee gait) walking, which entails greater power from all joints, including those on the paretic side. However, the peak extension power becomes nearly symmetric only at the hip (increasing from 55% to 85% of the contralateral value). The mechanical essence of hemiparetic gait, therefore, seems to be a matter of lower limb power asymmetry, not less than of absolute power deficit (15, 41). In the case of more symmetric paresis (e.g., in some cases of cerebral palsy), the hips, not the ankles, provide the main power output (63). In short, in central paresis, the foot loses its dominant propulsive role, compared to the hip and, to a minor extent, the knee, despite its capacity to increase power output in other contexts (e.g., crouched gait, walking faster or uphill). As a further consideration, plantar flexor power is needed to accelerate the body system forward during the step-to-step transition, more than it is allowed by the synchronous fall of the CoM. Negotiating body negative and positive accelerations may be troublesome in most cases of vestibular and cerebellar impairments and/or lower limb sensory deficits. Therefore, in the Authors' experience, attenuating and smoothing the “push-off” and transferring motor control from the ankle to proximal joints may flag hidden balance deficits.

Last, it is worth mentioning that the recent literature has looked for “global” parameters able to assess the quality and efficiency of walking in pathologic gait (4). In this regard, the CoM's mechanical energy changes and displacement (i.e., its three-dimensional trajectory) during the gait cycle are considered promising indexes. For example, vertical and lateral displacements of the CoM (64), the Margins of Stability (65) and the curvature peaks of the CoM's path (34) have been used as measures of dynamic stability during walking. Recently, in a sample of unilateral trans-femoral amputees, the contribution of the lower limb muscles and prosthesis to the CoM's accelerations and progression has been studied (66). In addition, methods for estimating CoM kinematics using low-cost instrumentation (e.g., inertial measurement units) are now available (67). Therefore, the present study provides further evidence linking the joint kinetics to CoM mechanics, aiming towards the clinical implementation of CoM-related measures and their application in longitudinal studies (68).

### 4.4 Clinical-evolutionary correlations

As a matter of speculation, the reasons for “renouncing” the power of the ankle during gait in central neurologic disorders may be related to the peculiarities of the human foot in hominoid evolution (69), mirroring the evolution of hominoid walking (70, 71). Bipedal animals, including apes, still retain a dominant propulsive role of the hip and knee extensor muscles: the same happens in humans, after a lesion of the central nervous system. Beyond many other anatomical differences from human feet, apes' feet do not benefit from the stiff “spring” made by the sagittal and transverse plantar arches (67, 72). During children's growth, adult-like plantar arch (73), kinematic and dynamic patterns of lower limb joints, including plantar flexion power (59, 74), are reached between ages 4 and 13, depending on the gait parameters analysed. The same holds for the pendulum-like motion of the body CoM (75). Walking in central paresis might thus be interpreted as a regression towards more primitive gait patterns, as per John Hughlings Jackson's fundamental interpretation of the “evolution and dissolution of the nervous system” (76–78). Loss of plantar flexion power during walking might be considered one of the hallmarks of this regression. Not surprisingly, visually inferring a decreased ankle push-off power has been suggested as a relevant goal in the clinical observation of gait (79, 80).

## Data Availability

The raw data supporting the conclusions of this article are available from the authors upon reasonable request.
